# Synthesis of Metal and Metal Oxide Nanoparticles by Flame Spray Pyrolysis and Safety Assessment

**DOI:** 10.3390/toxics14040330

**Published:** 2026-04-15

**Authors:** Ioanna Efthimiou, Yiannis Georgiou, Dimitris Vlastos, Stefanos Dailianis, Yiannis Deligiannakis, Maria Antonopoulou

**Affiliations:** 1 Department of Biology, Faculty of Sciences, University of Patras, GR-26500 Patra, Greece; dvlastos@upatras.gr (D.V.); sdailianis@upatras.gr (S.D.); 2 Department of Physics, University of Ioannina, GR-45110 Ioannina, Greece; yiannisgeorgiou@hotmail.com (Y.G.); ideligia@uoi.gr (Y.D.); 3 Department of Sustainable Agriculture, University of Patras, GR-30131 Agrinio, Greece

**Keywords:** nanoparticles, flame spray pyrolysis, CBMN, genotoxicity, cytotoxicity, humic acid

## Abstract

Zinc oxide (ZnO), silver (Ag) and titanium dioxide (TiO_2_) nanoparticles (NPs), are three of the most widely manufactured NPs, while composite NPs have gained popularity due to their enhanced properties. NP release in environmental matrices increases chances of bioavailability and subsequent impact on human health. The current study focuses on manufacturing, characterization and cyto-genotoxic assessment of Ag, ZnO/Ag, TiO_2_ and TiO_2_/Ag NPs with and without humic acids (HAs), aiming for a holistic approach that leads to a comprehensive profile of said NPs. It entails (a) the synthesis of the aforementioned NPs via single-nozzle Flame Spray Pyrolysis (SN-FSP); (b) the characterization of NPs (in powder form and in dispersion media) using Powder X-ray Diffraction (PXRD), Transmission Electron Microscopy (TEM) and Dynamic Light Scattering (DLS); and (c) the assessment of their genotoxicity and cytotoxicity against human lymphocytes in presence of two HAs, thus simulating actual environmental conditions, and without HAs, through the cytokinesis block micronucleus assay (CBMN) with cytochalasin-B. No genotoxicity was observed in any case, whereas cytotoxicity induction varied depending on the NP and the presence or absence of the two HAs. Therefore, it is indispensable to evaluate the toxic profile of NPs considering different environmental scenarios, while conducting an integrated characterization of NPs.

## 1. Introduction

Nanotechnology constitutes an emerging field these last decades, since engineered nanoparticles (NPs) are manufactured and used in a plethora of applications and products [1]. In fact, NPs have been utilized in electronics, cosmetics, food packaging and coatings, as well as in catalysis and biomedicine amongst others [2]. NPs, having a size at the range of 1 to 100 nm, possess significantly different physicochemical properties than their bulk counterparts, namely greater surface areas, high stability, reactivity, optical, thermal, and antimicrobial properties [3,4]. There are several categories of NPs such as fullerenes, polymeric and metal NPs [5].

Flame Spray Pyrolysis (FSP) constitutes one of the most scalable and affordable techniques for nanomaterials synthesis, allowing the production of many metal and metal oxide nanoparticles with specific physicochemical properties among others [6]. FSP possesses significant advantages compared to conventional manufacturing techniques of nanomaterials. Specifically, FSP leads to large scale production of NPs in a fast and continuous manner while showing unique reproducibility. The size and crystal structure of the nanomaterials produced can be regulated by proper adjustments in each case. Moreover, it does not lead to byproducts that need costly treatments for their disposal. It is noteworthy to mention that the high chemical purity of NPs produced via FSP, improves their biocompatibility and stability, leading potentially to significant applications in the health care sector [7]. In addition, FSP-produced nanomaterials have a wide range of applications in the field of environmental remediation due to their properties, including large surface area, good stability and high reactivity [8].

Metal and metal oxide NPs, including Ag, ZnO, and TiO_2_, comprise the largest part of the NP market due to their exceptional properties and numerous applications [9]. In fact, according to the Consumer Product Inventory, there are 443, 38 and 93 commercial products containing Ag, ZnO, and TiO_2_ NPs, respectively [10]. Ag NPs are particularly known for their excellent antimicrobial activity and have been used in textiles, food packaging, cosmetics, electronics, and healthcare products [11,12]. TiO_2_ and ZnO are transition metal oxides, exhibiting great photocatalytic activity under UV light and biocompatibility [13,14,15]. ZnO NPs have great semiconductor, optical and antimicrobial properties and have been utilized to produce electronics, textiles, and sunscreens [16,17]. TiO_2_ NPs find numerous applications in drug delivery, coating, catalysis, food packaging, personal care products and sunscreens [18,19,20,21].

Metal oxide-based composite NPs are gaining attention due to various potential applications, such as removal of hazardous pollutants [22,23], degradation of organic and inorganic compounds via photocatalysis [24] and antibacterial activity [25]. Namely, Ag/TiO_2_ and Ag/ZnO have been manufactured and used in textile fibres, leading to improvement of their photocatalytic self-cleaning and antimicrobial properties compared to their single counterparts [26,27,28]. Furthermore, enhanced antibacterial activity was exhibited by Ag/TiO_2_ nanocomposites due to the synergistic effect of nanosilver [29]. In addition, Ag-doped TiO_2_ NPs could find use in cancer treatment due to their reactive oxygen species (ROS)-generating potential via specific modifications that could result in the killing of cancer cells without harming normal cells [30,31].

Despite the several benefits in everyday life attributed to nanotechnology and its products, NPs will inevitably end up in different environmental matrices and thus the study of their potential effects on humans and the environment is imperative [32]. Ag, ZnO, and TiO_2_ NPs are among the most used metal NPs in agriculture, food, medicine, and several fields of industry [33,34,35]. Consequently, humans are systematically exposed to these NPs which could pose a significant health hazard [36]. Metal and metal oxide NPs have been found to have the most pronounced cytotoxic effects. Increased production of ROS, cell membrane damage and DNA damage consist some of their most well-known negative effects [37,38]. However, it should be considered that different synthesis methods can significantly affect the physicochemical properties of NPs [39,40] and naturally their impact in biota and the environment.

Despite the plethora of studies concerning the toxic profile of various NPs, other environmental factors should be considered since NPs will inevitably end up in different environmental matrices [41,42]. Natural organic matter (NOM) is omnipresent in the soil and aquatic environments and consists predominantly of humic acid (HA) as well as proteins, lipids, and polysaccharides amongst others [43,44,45]. HA can influence the mobility, transportation and bioavailability of many compounds including contaminants such as metals [46]. NPs released in the environment via manufacturing, usage and disposal will potentially adsorb to NOM. In fact, NP coating by HA can affect their surface properties and ability to adsorb toxic compounds [47]. It has been previously demonstrated that NOM could create a coating when it adsorbs on the surface of NPs leading to the formation of the eco-corona [48,49]. Thus, the media where NPs may be found as well as the ionic strength can impact their rate of agglomeration [50,51,52,53]. It is noteworthy to mention that NOM can have a different impact depending on the NP, either mitigating its toxicity by acting as a physical barrier and preventing its detrimental effects [54,55] or enhancing its toxicity by decreasing its agglomeration and facilitating its internalization [56].

Considering the aforementioned information and the potential risk of NPs spread in the environment, in addition to their impact on human health, the present study focuses on the investigation of the genotoxic and cytotoxic potential of novel Ag, ZnO/Ag, TiO_2_, and TiO_2_/Ag NPs in human lymphocytes with and without two HAs (HALP and LHA), using the cytokinesis block micronucleus (CBMN) assay. Thus, a well-characterized humic acid-like-polycondensate (HALP) [57] and a leonardite HA (LHA) obtained by the International Humic Substances Society (IHSS) [58] were used. HALP recreates the fundamental physicochemical parameters, such as charge, structural carboxy/phenolic content, and radicals, while lacking any adventitious ions—particularly Fe—that could induce adverse oxidative stress events [57]. CBMN assay has been widely used for the determination of the genotoxicity of various compounds including NPs [59,60] due to its simplicity and sensitivity. The frequency of micronuclei (MN) induction in the cytoplasm of interphase cells is determined to identify the potential genotoxic activity of the tested chemical [61,62]. In addition, the cytotoxic potential of the compound can be assessed as well using the cytokinesis block proliferation index (CBPI) [61,63]. Consequently, all NPs were fabricated using single-nozzle Flame Spray Pyrolysis (SN-FSP) [64], they were characterized via Powder X-ray Diffraction (PXRD), Transmission Electron Microscopy (TEM) and Dynamic Light Scattering (DLS) and assessed for their genotoxic and cytotoxic impact with and without HAs. A lack of genotoxicity was observed in all cases for single NPs and their mixtures with HAs. On the other hand, cytotoxic potential varied depending on the NP tested as well as the presence or absence of the two HAs. Therefore, the type of NP, its method of fabrication and the presence or absence of other environmental factors such as HAs can significantly affect and modify its toxic profile.

## 2. Materials and Methods

### 2.1. Chemicals and Reagents

Ag NPs, ZnO/Ag NPs, TiO_2_ NPs, and TiO_2_/Ag NPs were manufactured at the Department of Physics, at the University of Ioannina. The solvents employed in the experiments, namely xylene (CAS #: 1330-20-7, ACS grade) and acetonitrile with a purity of 99.5% (CAS #: 75-05-8, Reag. Ph Eur), were commercially sourced from Merck and used as received. For the synthesis of FSP particles, Titanium (IV) Isopropoxide (TTIP) (CAS #: 546-68-9) with a purity of 97% and noble metal–organic precursor was utilized. This noble metal precursor, the Silver (I) Acetylacetonate (CAS #: 15525-64-1), was procured from Alfa Aesar and boasted a purity level of 98%. Ham’s F-10 medium, fetal bovine serum (FBS) (Cat #: 16000044) and Phytohaemaglutinin (PHA) (Code: 10576015) were purchased from Gibco (London, UK), while HEPES (CAS #: 7365-45-9) was commercially supplied from Applichem (Darmstadt, Germany) and cytochalasin-B (Cyt-B) (CAS #: 14930-96-2) from Sigma-Aldrich Chemical Co. (St. Louis, MO, USA). All other chemicals and solvents were of the highest grade commercially available. The production of HALP took place via oxidative polymerization of gallic acid and protocatechuic acid in a 1:1 M ratio without use of co-catalyst according to Giannakopoulos et al. and its full characterization was reported in the same research paper [58]. Leonardite Humic Acid Standard (LHAS04) was purchased from the International Humic Substances Society (IHSS) (St. Paul, MN, USA) and used without further purification.

### 2.2. NP Preparation and Analysis

All NPs used were prepared using a SN-FSP reactor [64]. This is a scalable technology for the manufacturing of NPs with high crystallinity and purity at gram per hour rates; the process was conducted as previously described [65]. The analytical procedure for the preparation of Ag and ZnO/Ag NPs has been meticulously stated in our previous work [66]. For TiO_2_ production, a solution comprising TTIP, and then for TiO_2_/Ag production, a noble metal precursor (NM), in our case Silver (I) Acetylacetonate (61.00 × 10^−3^ M), is amalgamated with a solvent blend of xylene and acetonitrile (in a ratio of 2.2 to 1.0), maintaining a constant titanium concentration of 0.64 M. The proportion of Ag is 5 wt% relative to titanium, which is modulated by altering its concentration in the solvent mix. A liquid precursor solution containing both titanium and NM atoms is fed into the capillary tube at a rate of 5 mL min^−1^ using a syringe pump. During the SN-FSP procedure, a premixed methane and oxygen flame (2.5/5 L min^−1^ respectively) is ignited and maintained stable. The resultant particles are collected using a vacuum pump (Busch, Mink MM 1202 AV, Athens, Greece) and gathered on a glass fibre filter (ALBET-Lab Science, GF6, 25.7 cm, Dassel, Germany) positioned from the source, which means the distance of the nozzle to filter is 69 cm.

Powder X-ray Diffraction (PXRD) patterns were acquired utilizing a D8 Advance Bruker diffractometer (Billerica, MA, USA). This apparatus employs Cu Kα radiation (operating at 40 kV and 40 mA) and is fitted with a secondary beam graphite monochromator. The diffraction patterns were methodically recorded across a 2-theta (2Θ) range spanning from 10 to 90°, with incremental steps of 0.02° and a dwell time of 2 s per step.

Transmission Electron Microscopy (TEM) images were captured using a JEOL JEM2100 High-Resolution TEM microscope, operational at a voltage of 200 kV. For sample preparation, the specimens were initially dispersed in water and subjected to ultrasonication for a duration of 5 min. Subsequently, a droplet of this dispersion was carefully placed onto a copper grid coated with a carbon film and allowed to dry at ambient temperature.

### 2.3. Dynamic Light Scattering (DLS) and Zeta Potential

Determination of the particle size and polydispersity index of all NPs was performed with and without HALP and LHA in suspension. Specifically, suspensions of 20 μg mL^−1^ of Ag NPs, ZnO/Ag NPs, TiO_2_ NPs, and TiO_2_/Ag NPs were studied in (a) 2dH_2_O (double distilled H_2_O) and (b) the culture medium, pH 7 (6.5 mL of Ham’s F-10 medium, 1.5 mL of FBS and 0.3 mL of PHA). Moreover, NP combinations with HALP and LHA were studied at the highest doses (i.e., 20–32 μg mL^−1^ and 20–80 μg mL^−1^, respectively). NP agglomerate size was studied in 2dH_2_O following 20 min sonication (220 V, 50 Hz, 140 mA), whereas their size in culture medium was determined after 72 h (according to the protocol followed for the treatment of cell cultures). The zeta potential of NPs with and without HAs in 2dH_2_O was determined from their electrophoretic mobilities according to the Smoluchowski model. For the calculation of the agglomerate size in the culture medium, disposable small volume polystyrene cuvettes were used for the DLS measurements. In all other cases a clear disposable folded capillary zeta cell was used for the remaining DLS and zeta potential measurements. In all cases, a Malvern Zetasizer Nano series instrument was used.

### 2.4. Preparations of NP Working Solutions

Working solutions of single NPs and mixtures of NPs-HALP and NPs-LHA were prepared and maintained in 4 °C until use. Specifically, dispersion of 10 mg of each NP in 10 mL of HEPES buffer (0.01 M of HEPES in Milli-Q water, pH = 7 adjusted with small volumes of NaOH) was conducted for the preparation of stock solutions with a final concentration of 1 mg mL^−1^. Afterwards, stock solutions of NPs-HALP and NPs-LHA were prepared via the addition of 10 mg NPs-16 mg HALP and 10 mg NPs–40 mg LHA in 25 mL HEPES buffer, respectively (see Supplementary Materials Table S1). The concentrations were selected after preliminary experiments, while considering previous published work regarding similar research [59,60].

### 2.5. CBMN Assay in Human Lymphocytes

#### 2.5.1. Ethics Statement

The CBMN assay using human lymphocytes was carried out in accordance with international bioethics criteria, after the permission/approval of the Research Ethics Committee of the University of Patras (Ref. No. 11584/6 March 2018). After obtaining the written informed consent, two healthy non-smoking male individuals (less than 30 years), who were not exposed to radiation, were not under any drug treatment and did not have any viral infection in the recent past, were used as blood donors to establish whole blood lymphocyte cultures.

#### 2.5.2. CBMN Assay Application

CBMN assay was performed according to OECD 487 (2016). Two experimental replicates were performed using blood from two different donors. Each culture contained 6.5 mL of Ham’s F-10 medium, 1.5 mL fetal bovine serum (FBS), and 0.3 mL phytohaemagglutinin for the stimulation of cell division, in addition to 0.5 mL of whole blood from each donor. NP and NPs-HA working solutions were dispersed using a bath sonicator (220 V, 50 Hz, 140 mA) for 20 min and added to each sample 24 h after the culture initiation. Twenty h later, cytochalasin-B (Cyt-B, at a final concentration of 6 μg mL^−1^) was added in all cultures, to prevent cytokinesis (inhibition of actin polymerization) and allow nuclear division and the formation of binucleated (BN) cells with low baseline micronuclei (MN) frequency [67]. Cultures were incubated for 72 h (Thermo Scientific Incubator, Waltham, MA, USA), at 37 °C, under 5% CO_2_. After the end of the incubation period, cells were harvested and collected via centrifugation (1500 rpm, 10 min). They were treated with a mild hypotonic solution (Ham’s F10 medium:Milli-Q H2O, ratio 3:1) for 3 min at room temperature and subsequently fixated with a freshly prepared solution of methanol:acetic acid (ratio 5:1) in triplicate. Cell staining with 7% *v*/*v* Giemsa was the final step before further analysis.

The MNScore and MetaCyte system, based on the slide-scanning Metafer platform (MetaSystems, Altlussheim, Germany) was used for the scoring of MN in BN cells. For each experimental point at least 2000 BN cells with preserved cytoplasm were scored, according to well-established criteria [63,68]. Cytotoxicity determination was accomplished through the calculation of the cytokinesis block proliferation index (CBPI), after counting 1000 cells for each experimental point, using the equation:

CBPI = [N1 + N2 + 3(N3 + N4)]/N,

with N1, N2, N3, and N4 representing the numbers of cells with one, two, three and four nuclei, and N being the total number of cells [69].

### 2.6. Statistical Analysis

The results are expressed as mean ± standard error. The G-test for independence on 2 × 2 tables was used to perform the statistical analysis of the MN data. The chi-square test (χ^2^ test, *p* < 0.05) was used for the analysis of CBPI (Minitab^®^ 18 and IBM SPSS 19.0 Inc. software packages).

## 3. Results

### 3.1. Characterization of NPs

All NPs utilized were synthesized employing a SN-FSP reactor. This method represents a scalable approach for producing NPs with notable crystallinity and purity, achieving gram-per-hour production rates. The procedure adhered to the methodologies previously outlined [70]. More detailed analytical methods for fabricating TiO_2_ and TiO_2_/Ag NPs are thoroughly documented in our prior research [71].

#### 3.1.1. Powder X-Ray Diffraction (PXRD)

**Figure 1:**
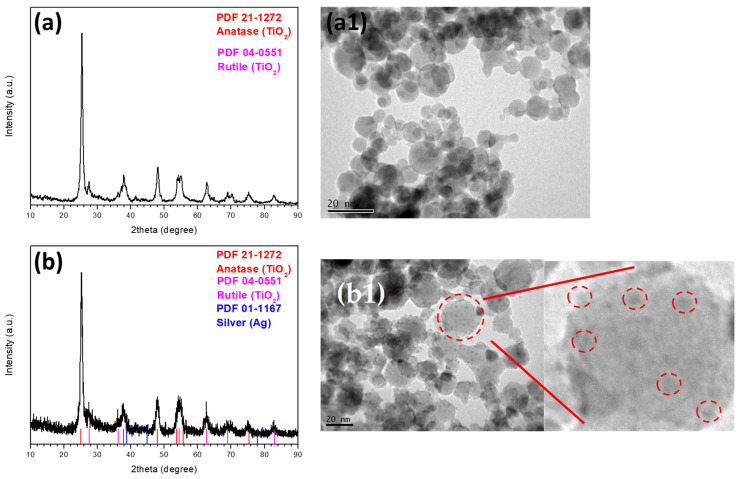
PXRD for (**a**) TiO_2_ and (**b**) TiO_2_/Ag NPs and TEM images for (**a1**) TiO_2_ and (**b1**) TiO_2_/Ag NPs with zoomed part that shows small (2–4 nm) Ag NPs homogenously dispersed in the TiO_2_ surface. The big red circle in b1 image shows the TiO_2_ NP surface, while the red lines show its enlarged version with the small circles representing Ag NPs in the surface of the TiO_2_ NP.

The PXRD analysis revealed distinct peaks at 2θ values of 25.3°, 37.8°, 48°, 53.9°, 62.7°, 70.3°, 75° and 83° which are indicative of the facets of the TiO_2_ anatase crystal structure. Peaks observed at 2θ values of 27.3°, 36°, 41°, 43°, 53°, 61°, and 70° are characteristic of the rutile crystal phase of TiO_2_. Overall, the PXRD patterns of the TiO_2_/Ag NPs did not exhibit significant variance from those of pure TiO_2_. No discernible PXRD peaks were attributed to Ag particles (Figure 1). This observation aligns with the TEM findings, where Ag particles were noted to be smaller than 2–4 nm, marked by the dashed circles in Figure 1b1, a dimension that typically yields weak diffraction peaks in PXRD.

#### 3.1.2. Transmission Electron Microscopy (TEM)

TEM assessments disclosed that the TiO_2_ NPs possess a spherical configuration, measuring 18 nm. Meanwhile, the TiO_2_/Ag NPs exhibited a co-agglomerated form, each measuring 17 nm in diameter (Figure 1), thereby corroborating the data obtained from PXRD analyses via the Scherrer equation. In the TiO_2_/Ag material the TEM data confirm the homogeneous dispersion of small Ag NPs tightly anchored on the TiO_2_ surface (Figure 1b1).

#### 3.1.3. Characterization of NP Working Solutions

The results from the DLS measurements are reported in Table 1. Low agglomeration rates were observed for Ag NPs and their mixtures with HAs in 2dH_2_O water, and they had high, negative zeta potential values. The average sizes of Ag NPs and their mixtures with HAs were slightly higher in the culture medium, with Ag NPs-LHA showing the greatest increase. ZnO/Ag NPs had an average particle size of 566.3 nm in 2dH_2_O, which was subsequently decreased in the presence of both HALP (206.6 nm) and LHA (169.5 nm). Negative and relatively high zeta potential values were recorded in all cases. A decrease in the average particle size was observed in the culture medium for ZnO/Ag NPs and ZnO/Ag NPs-LHA, whereas in ZnO/Ag NPs-HALP the size was slightly increased. According to the measurements in 2dH_2_O water, TiO_2_ NPs showed a high agglomeration rate with an average particle size of 1442 nm, and their surface was positively charged as demonstrated by the zeta potential (0.142 mV). However, their mixtures with both HAs had significantly lower average particle sizes, namely 485.7 nm and 377.1 nm for TiO_2_ NPs-HALP and TiO_2_ NPs-LHA, respectively. Negative and high zeta potential values were recorded for both mixtures with HAs (TiO_2_ NPs-HALP: −21.8 mV; TiO_2_ NPs-LHA: −48 mV). In the culture medium, TiO_2_ NPs, and their mixture with HALP had a smaller average particle size, whereas TiO_2_ NPs-LHA size was significantly increased. Regarding TiO_2_/Ag NPs average size, similar values were recorded for both single NPs and their mixtures with HAs in 2dH_2_O water, with the mixtures retaining slightly smaller values. All were highly stable as shown by the increased zeta potential values which were negative in every case. The sizes of TiO_2_/Ag NPs and their mixture with LHA were slightly higher in the culture medium, whereas TiO_2_/Ag NPs-HALP size was moderately decreased.

### 3.2. Genotoxicity in Treated Cells

Slightly higher MN frequencies were observed in lymphocytes treated with Ag NPs, Ag NPs-HALP and the highest concentration of Ag NPs-LHA (20 μg mL^−1^). A similar pattern was observed for ZnO/Ag NPs and their mixtures with HAs. On the other hand, lymphocyte cultures treated with TiO_2_ NPs and TiO_2_/Ag NPs with and without the two HAs had lower MN frequencies compared to the negative control. In all cases no statistically significant MN induction was recorded compared to the negative control, indicating a lack of genotoxic potential (see Supplementary Materials Table S2).

### 3.3. Cytotoxicity in Treated Cells

**Figure 2:**
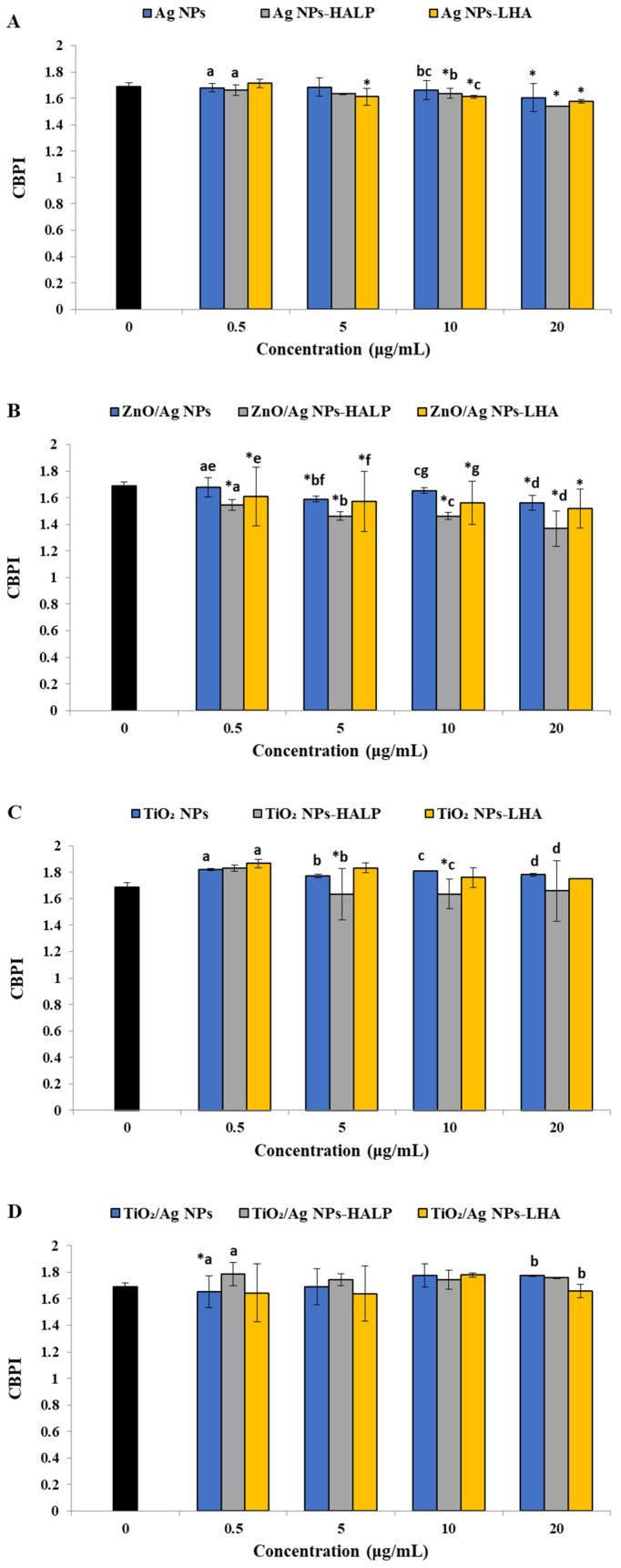
Cytotoxicity (in terms of CBPI values) of (**A**) Ag NPs, Ag NPs-HALP and Ag NPs-LHA; (**B**) ZnO/Ag NPs, ZnO/Ag NPs-HALP and ZnO/Ag NPs-LHA; (**C**) TiO_2_ NPs, TiO_2_ NPs-HALP and TiO_2_ NPs-LHA; (**D**) TiO_2_/Ag NPs, TiO_2_/Ag NPs-HALP and TiO_2_/Ag NPs-LHA in human lymphocytes. The results are mean ± SD from 2 independent experiments in each case. Values that share the same letter differ from each other. Asterisks (*) indicate significant difference from control in each case (Mann–Whitney u-test, *p* < 0.05).

Regarding Ag NPs, only the highest concentration (20 μg mL^−1^) exerted a statistically significant cytotoxic potential. Moreover, the two highest concentrations of Ag NPs-HALP (10–16 and 20–32 μg mL^−1^) and the three highest concentrations of Ag NPs-LHA (5–20, 10–40 and 20–80 μg mL^−1^) were deemed cytotoxic (Figure 2A). A significant reduction in the CBPI values was recorded for ZnO/Ag NPs at concentrations of 5 and 20 μg mL^−1^ compared to the respective value of the negative control. All concentrations of ZnO/Ag NPs mixtures with both HAs induced cytotoxicity (Figure 2B). A lack of cytotoxicity was observed for TiO_2_ NPs and their mixture with LHA, whereas TiO_2_ NPs-HALP was cytotoxic at 5–20 and 10–40 μg mL^−1^ (Figure 2C). Only the lowest concentration of TiO_2_/Ag NPs (0.5 μg mL^−1^) was cytotoxic against human lymphocytes, while TiO_2_/Ag NPs mixtures with HALP and LHA were not cytotoxic (Figure 2D).

#### Comparison of the Cytotoxic Potential of All NPs with and Without HAs

The tested NPs are ranked according to their cytotoxic potential with and without HALP and LHA in Table 2, going from higher to lower cytotoxicity. ZnO/Ag NPs are the most cytotoxic in all cases, followed by Ag NPs (for further details see Supplementary Materials Figure S1).

## 4. Discussion

Even though there is a plethora of scientific studies concerning different types of NPs, most of them focus on a specific topic such as their fabrication, characterization, applications in technological fields and/or toxicity. However, significantly fewer studies follow a more encompassing approach that entails several of those topics.

### 4.1. Average Size and Zeta Potential

The aggregation and colloidal state of metal NPs in addition to the rate of release of their ionic counterparts, depend largely on the properties and the characteristics of the environmental medium [50,72]. Generally, when zeta potential has an absolute value of 30 mV, NP colloidal stability is considered satisfactory and is retained via electrostatic repulsion [73].

In the case of Ag NPs, the sizes of single NPs and their mixture with HALP ranged at similar levels in 2dH_2_O and the culture medium. On the other hand, Ag NPs-LHA were more aggregated at the culture medium (464 nm) than the 2dH_2_O (111 nm). The different response concerning the degree of aggregation could be attributed to the different physicochemical characteristics of the HAs. In fact, coated Ag NPs demonstrated different hydrodynamic sizes and colloidal stability, when they interacted with different forms of NOM [74]. ZnO/Ag NPs degree of agglomeration, with and without HAs in 2dH_2_O, was higher compared to Ag NPs, and Ag NPs-HAs. The combination of the two NPs may potentially lead to this result. In the culture medium, ZnO/Ag NPs, and ZnO/Ag NPs-HALP demonstrated enhanced aggregation compared to Ag NPs as well as their mixtures with HALP, whereas ZnO/Ag NPs-LHA had a smaller particle size than Ag NPs-LHA. The latter is in accordance with the results of Gunsolus et al. (2015) [74]. TiO_2_ NPs average size in 2dH_2_O had a value of 1442 nm, which significantly decreased in the culture medium (523.3 nm). It has been previously demonstrated that the presence of serum in the culture medium could form a protein corona through binding to the NPs, leading to a reduction in size [75,76]. TiO_2_ NPs-HAs mixtures had significantly smaller average sizes in 2dH_2_O compared to TiO_2_ NPs. Similarly, Topuz et al. (2014) recorded the dispersing effect of humic and fulvic acid for TiO_2_ NPs, since NPs had a smaller size in combination with the organic matter than the single NP suspensions [77]. According to previous research, the increased stability of NPs paired with HAs is due to strong steric stabilization and adsorption of the HA on the surfaces of NPs enhancing their dispersion in solutions [78,79]. A slight reduction in average particle size was noticed for TiO_2_ NPs-HALP in the culture medium compared to the one in 2dH_2_O, whereas an inverse pattern was recorded for TiO_2_ NPs-LHA which could be due to physicochemical differences in the two HAs leading to different responses. Since the pH value of the cultures was neutral, the negatively charged HAs are able to adsorb on the NPs surface through carboxyl and phenolic groups [80]. TiO_2_-Ag NPs with and without HAs had small average sizes in both 2dH_2_O and the culture media. In almost all cases, TiO_2_-Ag NPs agglomeration with and without HAs was lower than TiO_2_ NPs and TiO_2_ NPs-HAs but higher than Ag NPs and Ag NPs-HAs.

Ag NPs had a high value of z-potential (−24.9 mV). Fernando and Zhou (2019) [81] studied the interactions between humic acids and Ag NPs, indicating that the addition of humic acid to negatively charged Ag NPs leads to a higher z-potential due to the adsorption of negatively charged HA molecules. Similarly, in our study single Ag NPs had a value of −24.9 mV, while mixtures Ag NPs-HALP and Ag NPs-LHA had values of −44.8 and −40.8 Mv, respectively (Table 1). According to Fernando and Zhou (2019) [81] Ag NPs adsorption on HA is due to the electrostatic stabilizing effect, via enhancing the negative charge on Ag NPs surface. Z-potential values of ZnO/Ag NPs and their mixtures with HAs were negative, but smaller than the ones of Ag NPs and their mixtures. Single TiO_2_ NPs were quite unstable, in contrast with the mixtures with HAs which appeared stable with quite high negative values. The presence of HAs and their binding to the NPs probably increased their stability. TiO_2_-Ag NPs with and without HAs were highly stable as demonstrated by their zeta potential values. These values (>25 mV) indicate that repulsive forces prevail against attraction forces, thus hindering agglomeration [82]. Moreover, TiO_2_-Ag NPs and their mixtures with HAs were more stable than both of their single counterparts with and without HAs.

### 4.2. Genotoxic and Cytotoxic Activity

Ag NPs

The Ag NPs studied did not lead to a statistically significant MN induction in any concentration, suggesting a lack of genotoxicity. Human lymphoblastoid cells TK6 treatment with Ag NPs (10–30 μg mL^−1^), resulted in increased MN frequencies only in concentrations higher than 20 μg mL^−1^ [83]. Furthermore, Ag NPs did not exert genotoxic activity neither against mouse lymphoma cells [84] nor in vivo in Sprague Dawley rats when the micronucleus assay was applied [85]. It has been proven that the same Ag NPs display a different ecotoxicological profile depending on the test media used, which renders the investigation of their toxic effects in different organisms and cell lines indispensable [86]. As far as their cytotoxic activity is concerned, Ag NPs had similar CBPI values with the negative control at the two lowest concentrations tested (0.5 and 5 μg mL^−1^). Souza et al. (2016) [87] observed an increase in cellular viability and proliferation when Ag NPs (size 10 nm) where given to hamster ovary cell line CHO-K1 at a concentration of 1.25 μg mL^−1^. On the other hand, buffer solutions, such as HEPES (4-(2-hydroxyethyl) piperazine-1-ethanesulfonic acid), have been widely used since they do not bind on many metal ions, including Zn [88]. However, since HEPES contains amino groups and Ag ions form stable amine complexes, it is suggested that HEPES potentially creates complexes with Ag ions. This hypothesis was confirmed by Mousavi et al. (2015) [89]. Following HEPES addition in a 5.0 μM Ag^+^ solution, electromotive force (emf) was reduced, indicating the diminution of free Ag^+^ ions concentration, resulting from Ag^+^ binding to the buffer. Nevertheless, CBPI reduction was statistically significant at the highest concentration tested (20 μg mL^−1^), pointing to cytotoxic potential. In a study of Limbach et al. (2007) [90], cells exhibited oxidative stress that was 25 times greater than the one caused by metal ions. The cellular membrane hinders the entrance of metal ions, exhibiting oxidative stress in controlled levels. On the other hand, metal NPs can easily enter the cytoplasm in a specific pH (which is defined as suitable for their dispersion) and diffuse the metal ions, which amplifies the induced cytotoxicity. Through this mechanism—known as ‘Trojan horse’—the particles are integrated from the cells and can interact with organelles and compounds causing ROS production [90,91]. ROS elevation affects the antioxidative response of the organism leading to oxidative stress, which is considered as the main mechanism behind Ag NP toxicity [92]. It has been previously recorded that Ag NPs were able to induce ROS production and lipid peroxidation against human lymphocytes, as well as modify the structural and functional properties of the cell membrane. Specifically, oxidation of unsaturated fatty acids and the decrease in the percentage of polar lipids in the cell can change the morphology of the cellular membrane and its permeability for certain compounds [93].

Ag NP mixtures with HAs did not induce genotoxic effects. On the other hand, combination of Ag NPs with the two HAs (HALP, LHA) did not inhibit single NP cytotoxicity, but enhanced it instead. In fact, CBPI reduction was observed for both Ag NPs-HALP and Ag NPs-LHA mixtures, which was statistically significant in the two highest concentrations (10–16, 20–32 μg mL^−1^) and the three highest concentrations (5–20, 10–40, 20–80 μg mL^−1^), respectively. HAs possess a negative charge because of the presence of multiple carboxylic and phenolic groups [94]. Humic compounds can act both as stabilizing and as reducing agents for metal NPs and metal ions, respectively [52]. The ability of humic substances to bind on a metal ion is relatively higher compared to their ability to create complexes with metals [95]. HA exhibits a gradual increase in the negative z-potential since the concentration increases due to the greater proton separation in higher concentrations. HA concentration will affect z-potential and conductivity, which in turn will impact the surface charge and conductivity of Ag NPs when these are combined. HA-Ag NPs interactions can increase Ag NPs stability in higher HAs concentrations. HA is able to bind to Ag NPs via two different mechanisms. The first mechanism is specialized proton attachment in particles’ active sites and the second one is governed by the properties of the double layer [96]. Similarly with HEPES impact on Ag^+^ binding, HAs addition to pH = 7.5 led to Ag^+^ binding at 9 ± 2% and 16 ± 1% on HAs SRFA (Suwannee River fulvic acid II, Cat. No. 2S101F) and SRHA (Suwannee River humic acid II, Cat. No. 2S101H), respectively, whereas at pH value 9 binding percentages significantly increased. Therefore, pH plays an important role at the capability and the binding rate of Ag^+^ on NOM [89]. Considering our experimental conditions (HEPES, pH = 7), it could be assumed that only a small portion of Ag^+^ from Ag NPs was bound on HAs, possibly affecting the cytotoxic impact. Furthermore, a specific interaction mechanism has been observed between Ag NPs and their ions with HAs. According to this, a reduction in the size of Ag NPs bound to HAs was observed, while Ag ions bound to HAs were reduced and formed new Ag NPs which were different from the initial NPs [97]. Consequently, the above mechanism leads potentially to increased Ag NPs cytotoxicity.

ZnO/Ag NPs

ZnO/Ag NPs showed slightly increased MN frequencies which were not however statistically significant in any case. A relatively small number of studies concerning ZnO/Ag NPs toxicity have been conducted, most of which are focused on their antimicrobial activity. Burlibaşa et al. (2020) [98] investigated the genotoxic potential of Ag-ZnO NPs in human lymphocytes in vitro and against Mus musculus BALB/c in vivo. Enhanced MN induction was reported in the first case, whereas no genotoxic effects were shown in vivo. Concerning their cytotoxic activity, CBPI reduction was observed which was statistically significant in 5 and 20 μg mL^−1^ (Figure 2B). ZnO/Ag NPs demonstrated dose-dependent effects against cancer cell lines (MCF7, HCT116, A549) regarding their viability [99]. In a study investigating the toxic effects of ZnO NPs mixtures with Ag ions against *Dapnia magna*, a synergistic action was observed when ZnO NP percentage was higher than Ag ions, whereas an antagonistic relationship prevailed when Ag ions were in a greater percentage than the NPs [100]. The synergistic action could be attributed to the disturbance of the physiology or metabolic processes, due to excessive Zn ion release or increased transport and accumulation through forming ZnO NPs-Ag complexes. Furthermore, in high ZnO NP concentrations, Ag ions can be adsorbed in their surface hydroxyl group, and subsequently be transported in the organism via ‘Trojan Horse’ mechanism, leading to an enhanced toxicity [101]. Azizi et al. (2016) [102] investigated ZnO/Ag NP cytotoxic activity against a kidney cell line (Vero cells, African Green Monkey kidney cell line) and reported that the increased cytotoxicity could be attributed to NPs’ small size, since it offers a higher contact surface to the cells. A similar phenomenon may have taken place in the present study since NP size was 19.6 and 10.3 nm for ZnO and Ag NPs, respectively [66].

Regarding ZnO/Ag NPs mixtures with HAs, there was no genotoxicity induction in any case. On the other hand, all concentrations tested showed a statistically significant increase in cytotoxic activity. According to the previous references, there was potentially a synergistic action between NPs and HAs. In addition, taking into account the mechanism leading to the formation of new Ag NPs during the interaction of HAs with Ag^+^, the elevated cytotoxicity against human lymphocytes could be explained, since NPs-HAs mixtures demonstrated a more pronounced cytotoxic effect compared to the single ZnO/Ag NPs.

TiO_2_ NPs

Regarding the results of TiO_2_ NPs’ toxic profile, no genotoxic and/or cytotoxic effects were exerted against human lymphocytes. According to the existing literature, there have been conflicting results concerning the impact of TiO_2_ NPs against different cells. In fact, cytotoxic and genotoxic effects have been induced due to said NPs [103,104,105], whereas no or negligible toxic effects were recorded in various cell lines [15,106,107]. In addition, different cell lines, assays and tested concentrations lead to varied responses. Ghosh et al. (2017) [108] examined the genotoxic activity of three types of TiO_2_ NPs, i.e., anatase, rutile and their mixture against bronchial epithelial (16-HBE) cells. Even though a dose-dependent toxicity was recorded for all three NPs via the comet assay, no statistically significant MN induction was found when the CBMN assay was applied. According to Kansara et al. (2015) [109] TiO_2_ NPs were genotoxic only above the concentration of 75 μg mL^−1^. Furthermore, MN frequencies in TK6 cells treated with TiO_2_ NPs were comparable with the ones of the negative control [110]. A lack of significant genotoxic effects was demonstrated against human endothelial lung cells BEAS-2B and an intestinal cell model, which were treated with TiO_2_ NPs [111,112]. Similarly, cytotoxic activity of TiO_2_ NPs against 16-HBE cells was statistically significant only at 25 μg mL^−1^ [108]. Nanosized anatase and rutile did not affect the CBPI, showing a lack of cytotoxicity against BEAS 2B cells [113]. When the CBMN assay was applied in TK6 cells for the assessment of TiO_2_ NPs cytotoxic potential, similar CBPI values were obtained for cultures treated with said NPs and non-treated ones, indicating a lack of cytotoxicity [110]. TiO_2_ NPs (80% anatase, 20% rutile) were tested for their cytotoxic activity against SH-SY5Y cells via 3-(4,5-dimethylthiazol-2-yl)-2,5-diphenyl tetrazolium bromide (MTT) and neutral red uptake assays, at concentrations ranging from 20 to 150 μg mL^−1^. No significant reduction in cell viability was observed [114]. The different crystalline forms of TiO_2_, namely anatase and rutile, could have a different impacts since anatase NPs are considered more biologically active [115,116]. Moreover, the manufacturing method used leads to different physical and chemical properties of NPs, which affects their final toxicity [117,118].

A similar pattern was recorded for TiO_2_ NPs-HAs mixtures concerning their genotoxic potential, given that no significant MN induction was observed in any case. However, TiO_2_ NPs-HALP was cytotoxic in two concentrations, whereas TiO_2_ NPs-LHA did not exert cytotoxicity compared to the control culture. TiO_2_ NPs can be stabilized via the introduction of organic matter, which is the case in the present study as showcased by the reduced size of NPs-HALP mixtures leading to increased bioavailability and ultimately higher cytotoxicity [119]. On the other hand, LHA favoured the aggregation of TiO_2_ NPs in the culture medium—as shown by the results of average particle size—which may have contributed to the lack of toxic effects against human lymphocytes. Moreover, different types of organic matter could lead to different outcomes in toxicity, as previously shown. In fact, mixtures of humic acid with TiO_2_ NPs exerted a higher phototoxicity than mixtures of Tannic acid with TiO_2_ NPs at 10 ppm but lower at 40 ppm [119]. Kansara et al. (2020) [120] demonstrated the protective effect of HA against TiO_2_ NP toxicity on zebrafish embryo development. HA has been found to act as a ROS scavenger or a physical barrier between NPs and the cell, reducing the negative effects of the NPs [121]. Furthermore, organic compounds including humic acid and proteins, are able to form an “eco-corona” around TiO_2_ NPs’ surface [122]. Humic acid could also act as an antioxidant against ROS, [123] which was the case in a study by Lin et al. (2012) [124] where TiO_2_ NP toxicity against algal cells was alleviated in the presence of HA via oxidative stress reduction.

TiO_2_/Ag NPs

As far as TiO_2_/Ag NPs are concerned, no genotoxic potential was recorded, while significant cytotoxicity was exerted only at the lowest concentration tested. Composite NPs, including TiO_2_/Ag NPs, have not been thoroughly tested as far as their genotoxic and cytotoxic impact is concerned. Most studies focus on their beneficial properties and various applications, which is also the case for TiO_2_/Ag NPs [125]. The only study examining the genotoxic effects of Ag-doped TiO_2_ NPs thus far was conducted by Mahjoubian et al. (2021) [126]. The genotoxic activity of Ag-doped TiO_2_ NPs against zebrafish (*Danio rerio*) was assessed, and an increase was recorded in micronuclei and nuclear abnormalities compared to the control group. However, it is well known that genotoxic effects vary in different organisms, cell lines and test systems. The cytotoxicity of TiO_2_/Ag NPs has been assessed by a few studies. Interestingly, it was found that Ag-doped TiO_2_ NPs induced cytotoxic damage against cancer cells including human liver (HepG2), human lung (A549) and breast (MCF-7) cancer cells, while lacking significant toxicity against normal cells namely human lung fibroblasts and primary rat hepatocytes [127]. A similar pattern was observed when the cytotoxicity of TiO_2_/Ag NPs was evaluated against Caco-2 colorectal epithelial adenocarcinoma cells and BJ normal human skin fibroblasts [128]. The viability of TiO_2_/Ag-treated BJ cell cultures did not decrease, except for the highest concentration tested, i.e., 200 μg mL^−1^, whereas the same NPs led to a significant loss of viability on Caco-2 cells at concentrations as low as 10 μg mL^−1^.

TiO_2_/Ag NP mixtures with HAs did not induce genotoxic or cytotoxic effects against the human lymphocytes. Even though no relevant literature can be found, it can be inferred that HAs had a protective role against NP toxicity in this case.

### 4.3. NP-Biocorona

Even though NPs’ biological effects are mostly ascribed to their oxidative action, their interactions with cellular structures and/or various biomolecules can lead to the formation of a nanoparticle-biocorona (BC) which is considered a dynamic, constantly evolving structure, since biomolecules are being adsorbed and replaced on the NP surface [129]. Specifically, it has been reported that the formation of an external ‘biolayer’ in the extracellular environment could alter the size, shape, and surface properties of the NP, creating a ‘biological identity’ which is different than the NP’s initial ‘synthetic identity’ [130].

According to the results of the present study, NP effects against the cell culture used could be explained by the formation of BC. It has been proven that cell culture media can significantly affect biomolecules adsorption on NPs. Maiorano et al. (2010) [131] reported that the cell culture medium can impact the BC composition on NPs and the cellular response alike. NPs smaller size in the cell culture medium—compared to their respective size in 2dH_2_O—could be attributed to the BC formation, which has been proven to modify NP size, since the biomolecules which are present in the medium cover NPs, reducing agglomeration [130]. Charged NPs [132] and hydrophobic surfaces [75] can lead to significant protein adsorption, since both entropy (hydrophobic effect) and enthalpy (charge interaction) favour adsorption. Similarly, the metal NPs of the present study possess surface charge, rendering the formation of BC feasible. BC composition varies depending on the biological fluid that NPs are in, and thus affecting their final size, which could explain the smaller sizes of the studied NPs in the culture medium [131].

The ability or inability of the NPs to enter the cell has been the focus of intensive research during the last years [133,134,135]. Zhu et al. (2013) [136] suggested that NPs adsorption is mostly a size-dependent process in eukaryotic cells due to the well-designed intracellular mechanism. Nevertheless, it has been stated that NP hydrophobicity could play a significant role on the aforementioned process [137]. Specifically, phospholipids which possess a hydrophilic head and two hydrophobic tails, could easily adsorb on NPs’ surface via hydrophobic and electrostatic interactions, [138] leading to BC formation. Through the previous process, endocytosis and autophagy could be facilitated [139]. In fact, Ag NPs in presence and absence of BC on their surface were able to provoke a dose-dependent cytotoxicity in lung epithelial cells and aortic endothelial cells of rats through endocytosis which is followed by the loss of PC [140]. Conversely, biomolecule adsorption on the NP surface could modify the proteins’ structure, leading to their denaturation [141,142,143].

Accordingly, NOM adsorption on the NP surface could lead to the formation of a layer called NOM-corona (NC), imparting new properties to the NPs [144]. NC constitutes the essential interface that regulates NPs’ surface characteristics (e.g., charge, adsorption capacity), their environmental behaviour (e.g., aggregation, dispersion) and their biological interaction (e.g., toxicity, bioavailability) [145]. The HAs of the present study (HALP, LHA) acted either protectively or enhanced single NP-mediated toxicity. Considering the results of the present study, it is apparent that the effect exerted by the corona varies depending on the different NPs and media (cell culture, presence of HAs), which can lead to the formation of a different type of corona and favour the uptake of specific surface biomolecules, enhancing or reducing the toxicity of the studied NPs. Consequently, the study of the corona formation on NPs is deemed as a fundamental future research focus [145,146].

## 5. Conclusions

Despite the extended scientific research concerning NPs, most studies focus on a specific factor and/or parameter, e.g., the synthesis method, the physicochemical characteristics and morphology and/or the potential applications. Even though each study enriches the scientific database and fills the knowledge gaps still existing in the field of nanotechnology, the present study aimed at a holistic approach. It is the first time the specific NPs (Ag, ZnO/Ag, TiO_2_, and TiO_2_/Ag), manufactured via SN-FSP, were examined through a CBMN assay regarding their cyto-genotoxic potential in human lymphocytes in presence of two humic acids, i.e., HALP and LHA. They did not exert any genotoxic potential in the concentrations and experimental conditions tested. However, the cytotoxicity exerted varied depending on the NP and the presence or absence of the two HAs. Consequently, the inclusion of NPs’ manufacturing method, their thorough characterization, and the assessment of their toxic profile considering different environmental factors should be undertaken to acquire robust and reliable results that will enhance our knowledge and understanding of NPs’ potential toxicity. In addition, NP risk assessment is a critical step, so that regulatory agencies can establish and enforce appropriate standards and policies regarding their applications in various fields in addition to protecting human health in relation to their environmental fate.

## Figures and Tables

**Table 1:** Particle size of NPs with and without HAs (HALP, LHA), zeta potential and polydispersity index in 2dH_2_O (A) and particle size and polydispersity index in the culture medium (B).

A	Z-Average Aggregate Particles Size (nm)	Ζeta Potential (mV)	Polydispersity Index
2dH_2_O			
Ag NPs			
Ag NPs 20 μg mL^−1^	110.4 ± 3.828	−24.9 ± 0.153	0.208 ± 0.020
Ag NPs-HALP (20–32 μg mL^−1^)	86.67 ± 1.249	−44.8 ± 0.971	0.335 ± 0.045
Ag NPs-LHA (20–80 μg mL^−1^)	111.2 ± 3.153	−40.8 ± 1.75	0.459 ± 0.026
ZnO/Ag NPs			
ZnO/Ag NPs 20 μg mL^−1^	566.3 ± 109.7	−17.2 ± 1.68	0.651 ± 0.059
ZnO/Ag NPs-HALP (20–32 μg mL^−1^)	206.6 ± 14.35	−22.1 ± 1.43	0.585 ± 0.034
ZnO/Ag NPs-LHA (20–80 μg mL^−1^)	169.5 ± 24.17	−33.6 ± 2.75	0.446 ± 0.146
TiO_2_ NPs			
TiO_2_ NPs 20 μg mL^−1^	1442 ± 264.9	0.142 ± 0.316	0.450 ± 0.083
TiO_2_ NPs-HALP (20–32 μg mL^−1^)	485.7 ± 14.60	−21.8 ± 2.64	0.466 ± 0.017
TiO_2_ NPs-LHA (20–80 μg mL^−1^)	377.1 ± 65.75	−48.0 ± 0.351	0.492 ± 0.074
TiO_2_-Ag NPs			
TiO_2_-Ag NPs 20 μg mL^−1^	430.3 ± 63.05	−39.8 ± 11.9	0.439 ± 0.032
TiO_2_-Ag NPs-HALP (20–32 μg mL^−1^)	345.8 ± 28.32	−68.3 ± 3.10	0.428 ± 0.011
TiO_2_-Ag NPs-LHA (20–80 μg mL^−1^)	382.6 ± 10.03	−51.8 ± 1.06	0.448 ± 0.025
B			
Culture medium			
Ag NPs			
Ag NPs 20 μg mL^−1^	174.2 ± 5.393	-	0.273 ± 0.015
Ag NPs-HALP (20–32 μg mL^−1^)	114.9 ± 1.735	-	0.407 ± 0.021
Ag NPs-LHA (20–80 μg mL^−1^)	464.4 ± 144.4	-	0.510 ± 0.144
ZnO/Ag NPs			
ZnO/Ag NPs 20 μg mL^−1^	314.2 ± 134.4	-	0.320 ± 0.105
ZnO/Ag NPs-HALP (20–32 μg mL^−1^)	331 ± 92.44	-	0.388 ± 0.061
ZnO/Ag NPs-LHA (20–80 μg mL^−1^)	127.4 ± 6.866	-	0.457 ± 0.088
TiO_2_ NPs			
TiO_2_ NPs 20 μg mL^−1^	523.3 ± 233.9	-	0.568 ± 0.142
TiO_2_ NPs-HALP (20–32 μg mL^−1^)	345.0 ± 72.87	-	0.428 ± 0.052
TiO_2_ NPs-LHA (20–80 μg mL^−1^)	922.8 ± 255.2	-	0.766 ± 0.123
TiO_2_-Ag NPs			
TiO_2_-Ag NPs 20 μg mL^−1^	453.0 ± 190.1	-	0.769 ± 0.220
TiO_2_-Ag NPs-HALP (20–32 μg mL^−1^)	277.2 ± 78.54	-	0.499 ± 0.127
TiO_2_-Ag NPs-LHA (20–80 μg mL^−1^)	452.2 ± 86.65	-	0.499 ± 0.015

**Table 2:** Ranking of the tested NPs with and without HALP and LHA according to their cytotoxic potential.

	Cytotoxicity
**NPs**	ZnO/Ag > Ag > TiO_2_/Ag > TiO_2_
**NPs-HALP**	ZnO/Ag > Ag > TiO_2_ > TiO_2_/Ag
**NPs-LHA**	ZnO/Ag > Ag > TiO_2_/Ag > TiO_2_

## Data Availability

The original contributions presented in this study are included in the article/Supplementary Materials. Further inquiries can be directed to the corresponding authors.

## Supplementary Materials

The following supporting information can be downloaded at: https://www.mdpi.com/article/10.3390/toxics14040330/s1, Table S1: Working stock solutions and concentrations tested in each case. In the case of NPs-HAs the first number corresponds to NPs and the second to the HAs’ concentration respectively; Table S2: MN frequencies expressed as number of MN (‰) ± standard error per 2000 binucleated cells per experimental point, in cultured human lymphocytes treated with different concentrations (μg mL^−1^) of (A) Ag NPs, Ag NPs-HALP and Ag NPs-LHA, (B) ZnO/Ag NPs, ZnO/Ag NPs-HALP and ZnO/Ag NPs-LHA, (C) TiO_2_ NPs, TiO_2_ NPs-HALP and TiO_2_ NPs-LHA, (D) TiO_2_/Ag NPs, TiO_2_/Ag NPs-HALP and TiO_2_/Ag NPs-LHA; Figure S1: Cytotoxicity (in terms of CBPI values) of (A) Ag NPs, ZnO-Ag NPs, TiO_2_ NPs, and TiO_2_/Ag NPs, (B) Ag NPs-HALP, ZnO/Ag NPs-HALP, TiO_2_ NPs-HALP, and TiO_2_/Ag NPs-HALP, (C) Ag NPs-LHA, ZnO/Ag NPs-LHA, TiO_2_ NPs-LHA, and TiO_2_/Ag NPs-LHA in human lymphocytes. The results are mean ± SD from 2 independent experiments in each case. Values that share the same letter differ from each other (Mann–Whitney u-test, *p* < 0.05).

## Abbreviations

The following abbreviations are used in this manuscript:

NP	Nanoparticle
SN-FSP	Single-nozzle Flame Spray Pyrolysis
PXRD	Powder X-ray Diffraction
TEM	Transmission Electron Microscopy
DLS	Dynamic Light Scattering
HAs	Humic acids
CBMN	Cytokinesis Block Micronucleus
MN	Micronuclei
BN	Binucleated
CBPI	Cytokinesis Block Proliferation Index
