# Supplementary material for: Synthesis of Metal and Metal Oxide Nanoparticles by Flame Spray Pyrolysis and Safety Assessment
Source: Toxics. 2026 Apr 15;14(4):330. doi: 10.3390/toxics14040330 (PMC13119571; doi:10.3390/toxics14040330)
Supplement: Supplementary file 1 [file toxics-14-00330-s001.zip › Supplementary Information.pdf]

Synthesis of Metal and Metal Oxide Nanoparticles by Flame Spray Pyrolysis and Safety Assessment

Ioanna Efthimiou [1,*], Yiannis Georgiou [2], Dimitris Vlastos [1], Stefanos Dailianis [1], Yiannis Deligiannakis [2] and Maria Antonopoulou [3,*]

[1] Department of Biology, Faculty of Sciences, University of Patras, GR-26500 Patra, Greece; dvlastos@upatras.gr (D.V.); sdailianis@upatras.gr (S.D.)
[2] Department of Physics, University of Ioannina, GR-45110 Ioannina, Greece; yiannisgeorgiou@hotmail.com (Y.G.); ideligia@uoi.gr (Y.D.)
[3] Department of Sustainable Agriculture, University of Patras, GR-30131 Agrinio, Greece
* Correspondence: iefthimiou@upatras.gr (I.E.); mantonop@upatras.gr (M.A.)

Table S1. Working stock solutions and concentrations tested in each case. In the case of NPs-HAs the first number corresponds to NPs and the second to the HAs' concentration respectively.

Working solution	Concentration tested (μg mL[-1])			
NPs	0.5	5	10	20
NPs-HALP	0.5 - 0.8	5 - 8	10 - 16	20 - 32
NPs-LHA	0.5 - 2	5 - 20	10 - 40	20 - 80

Table S2. MN frequencies expressed as number of MN (‰) ± standard error per 2000 binucleated cells per experimental point, in cultured human lymphocytes treated with different concentrations (μg mL[-1]) of **(A)** Ag NPs, Ag NPs-HALP and Ag NPs-LHA, **(B)** ZnO/Ag NPs, ZnO/Ag NPs-HALP and ZnO/Ag NPs-LHA, **(C)** TiO_2 NPs, TiO_2 NPs-HALP and TiO_2 NPs-LHA, **(D)** TiO_2/Ag NPs, TiO_2/Ag NPs-HALP and TiO_2/Ag NPs-LHA.

A	Concentration (μg mL[-1])	Ag NPs MN (‰)	p-value	Concentration (μg mL[-1])	Ag NPs-HALP MN (‰)	p-value
	0	3.8±1.0		0	3.8±1.0	
	0.5	3.5±0.7	-	0.5 + 0.8	7.0±1.4	0.141
	5	5.5±0.1	0.413	5 + 8	3.5±2.1	-
	10	4.0±0.1	0.919	10 + 16	4.5±3.5	0.727
	20	5.0±0.1	0.556	20 + 32	4.5±2.1	0.727
	**Concentration (μg mL[-1])**	**Ag NPs MN (‰)**		**Concentration (μg mL[-1])**	**Ag NPs-LHA MN (‰)**	**p-value**
	0	3.8±1.0		0	3.8±1.0	
	0.5	3.5±0.7		0.5 + 2	3.5±0.7	-

5	5.5±0.1		5 + 20	3.5±0.7	-
10	4.0±0.1		10 + 40	3.5±0.7	-
20	5.0±0.1		20 + 80	5.0±2.8	0.556

B	Concentration (µg mL^{-1})	ZnO/Ag NPs MN (‰)	p-value	Concentration (µg mL^{-1})	ZnO/Ag NPs-HALP MN (‰)	p-value
	0	3.8±1.0		0	3.8±1.0	
	0.5	6.0±1.4	0.297	0.5 + 0.8	4.5±0.7	0.727
	5	4.5±0.1	0.727	5 + 8	5.5±4.9	0.413
	10	5.0±0.1	0.556	10 + 16	4.5±4.9	0.727
	20	6.0±0.1	0.297	20 + 32	5.0±4.2	0.556
	**Concentration (µg mL^{-1})**	**ZnO/Ag NPs MN (‰)**		**Concentration (µg mL^{-1})**	**ZnO/Ag NPs-LHA MN (‰)**	**p-value**
	0	3.8±1.0		0	3.8±1.0	
	0.5	6.0±1.4		0.5 + 2	2.5±2.1	-
	5	4.5±0.1		5 + 20	3.0±2.8	-
	10	5.0±0.1		10 + 40	1.5±0.7	-
	20	6.0±0.1		20 + 80	4.5±2.1	0.727

C	Concentration (µg mL^{-1})	TiO_2 NPs MN (‰)	Concentration (µg mL^{-1})	TiO_2 NPs-HALP MN (‰)
	0	3.8±1.0	0	3.8±1.0
	0.5	2.0±0.0	0.5 + 0.8	3.0±1.4
	5	3.0±0.1	5 + 8	2.0±0.0
	10	2.5±0.1	10 + 16	2.5±0.7
	20	1.5±0.1	20 + 32	2.5±0.7
	**Concentration (µg mL^{-1})**	**TiO_2 NPs MN (‰)**	**Concentration (µg mL^{-1})**	**TiO_2 NPs-LHA MN (‰)**
	0	3.8±1.0	0	3.8±1.0
	0.5	2.0±0.0	0.5 + 2	2.0±0.0
	5	3.0±0.1	5 + 20	2.5±0.7
	10	2.5±0.1	10 + 40	2.5±2.1
	20	1.5±0.1	20 + 80	2.5±0.7

D	Concentration (µg mL^{-1})	TiO_2/Ag NPs MN (‰)	Concentration (µg mL^{-1})	TiO_2/Ag NPs-HALP MN (‰)
	0	3.8±1.0	0	3.8±1.0
	0.5	2.5±0.7	0.5 + 0.8	3.0±0.0
	5	2.0±0.1	5 + 8	2.0±0.0
	10	1.5±0.1	10 + 16	2.0±0.0
	20	2.5±0.1	20 + 32	2.5±0.7

Concentration (μg mL-1)	TiO_2/Ag NPs MN (‰)	Concentration (μg mL-1)	TiO_2/Ag NPs-LHA MN (‰)
0	3.8±1.0	0	3.8±1.0
0.5	2.5±0.7	0.5 + 2	2.0±1.4
5	2.0±0.1	5 + 20	2.5±0.7
10	1.5±0.1	10 + 40	1.0±0.0
20	2.5±0.1	20 + 80	3.0±1.4

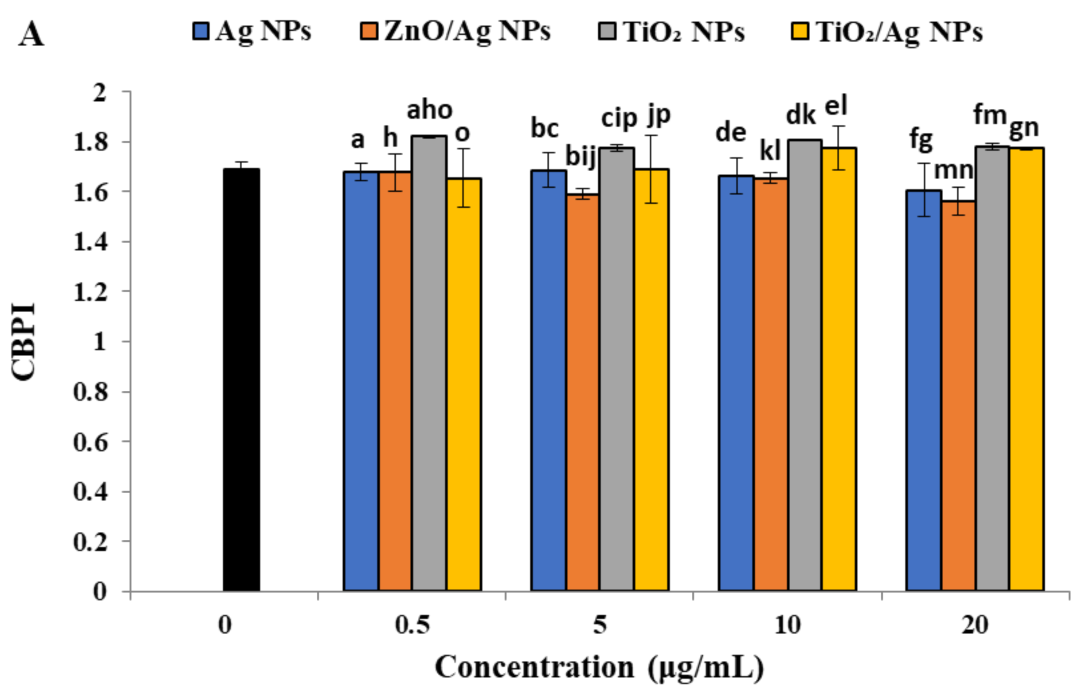

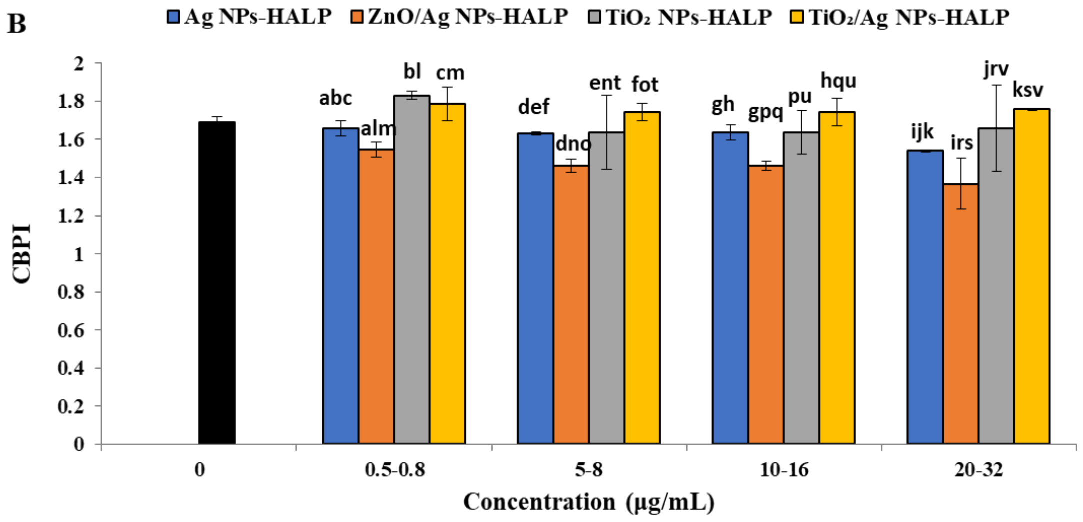

C

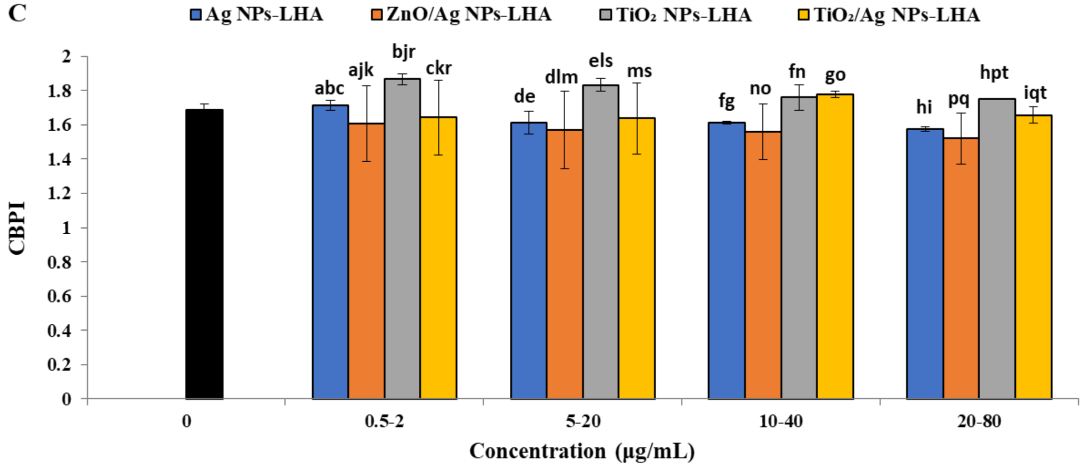

Figure S1. Cytotoxicity (in terms of CBPI values) of (A) Ag NPs, ZnO-Ag NPs, TiO_2 NPs, and TiO_2/Ag NPs, (B) Ag NPs-HALP, ZnO/Ag NPs-HALP, TiO_2 NPs-HALP, and TiO_2/Ag NPs-HALP, (C) Ag NPs-LHA, ZnO/Ag NPs-LHA, TiO_2 NPs-LHA, and TiO_2/Ag NPs-LHA in human lymphocytes. The results are mean ± SD from 2 independent experiments in each case. Values that share the same letter differ from each other (Mann–Whitney u-test, $p < 0.05$).
